# Combination of betulinic acid and chidamide inhibits acute myeloid leukemia by suppression of the HIF1α pathway and generation of reactive oxygen species

**DOI:** 10.18632/oncotarget.21889

**Published:** 2017-10-16

**Authors:** Hongyu Zhang, Ling Li, Min Li, Xiaodong Huang, Weiguo Xie, Wei Xiang, Paul Yao

**Affiliations:** ^1^ Department of Hematology, Peking University Shenzhen Hospital, Shenzhen 518036, P.R. China; ^2^ Department of Pediatrics, Maternal and Child Health Care Hospital of Hainan Province, Haikou 570206, P.R. China; ^3^ Institute of Burns, Tongren Hospital of Wuhan University, Wuhan 430060, P.R. China

**Keywords:** AHR, AML, HIF1α, reactive oxygen species, VEGF

## Abstract

Acute myeloid leukemia (AML) is a heterogeneous disorder of the hematopoietic system with no common genetic “Achilles heel” that can be targeted. Most patients respond well to standard therapy, while a majority relapse, and development of an effective therapy for AML patients is still urgently needed. In this study, we demonstrated that betulinic acid (BA) significantly increased Aryl hydrocarbon receptor (AHR) expression through demethylation on the AHR promoter in AML cells, and the increased AHR expression interacts with and sequesters ARNT, subsequently suppressing hypoxia-inducible factor-1α (HIF1α) pathway. We also found that histone deacetylase inhibitor chidamide (CDM) treatment significantly increased p300 over-acetylation in AML cells with dissociation of p300 with HIF1α, and subsequently suppressed the HIF1α pathway. Further investigation showed that BA/CDM combination additively increased generation of reactive oxygen species (ROS) with DNA damage, apoptosis and mitochondrial dysfunction. Also, BA/CDM combination additively suppressed the HIF1α pathway with decreased VEGF expression. *in vivo* mice study showed that BA/CDM combination significantly suppressed AML tumor growth, and overexpression of SOD2 and a constitutive HIF1α (HIF1C) completely diminished this effect. We conclude that a BA/CDM combination inhibits AML tumors through ROS over-generation and HIF1α pathway suppression. This is the first time we have shown the potential effect and possible mechanism of BA and CDM on the inhibition of AML tumor growth.

## INTRODUCTION

Acute myeloid leukemia (AML) is a heterogeneous disorder of the hematopoietic system with no common genetic “Achilles heel” that can be targeted [1]. It is caused by a number of genetic alterations and is characterized by uncontrolled cell proliferation, escape from apoptosis and blockage of myeloid differentiation [2, 3]. AML is mainly diagnosed in elder individuals within the range of 60-65 years old. The standard treatment for most AML patients often involves the use of 2 chemotherapy drugs, cytarabine (ara-C) and an anthracycline drug such as daunorubicin or idarubicin. Even though most patients respond well to therapy, a majority relapse [4]. In this case, development of an effective therapy for AML patients is still urgently needed [5].

Betulinic acid (BA) is a natural product that is derived from plant sources and has been characterized as a highly selective inhibitor of human melanoma cell [6] and tumor growth [7] through induction of apoptosis [8]. Also, BA can induce apoptosis on leukemia cells [9, 10], while the detailed mechanism of tumor inhibition still needs to be fully understood. Histone deacetylase (HDAC) inhibitors (HDACi) have been reported to be a class of antileukemic agents due to their promising effects on cell differentiation, cell cycle arrest, and apoptosis in human leukemic cells, but have much less of an effect in normal cells [11]. Chidamide (CDM, CS055) is a novel benzamide-type HDACi, a synthetic analogue of MS-275, and is currently in clinical trials for leukemia in China [7, 12]. CDM can induce significant cell-cycle arrest, resulting in the inhibition of cell proliferation and apoptosis in leukemia cells [13]. Recently, a striking report showed a complete molecular remission in a relapsed and refractory AML patient using a Chidamide-based protocol, indicating that CDM may be a good candidate for AML treatment [14], while the detailed mechanism is still unknown. Recently, we have found that combination of BA and CDM (BA/CDM) inhibits EBV (Epstein-Barrvirus) replication through generation of ROS (reactive oxygen species) in EBV-associated tumor cells [15], and we suppose that BA/CDM combination may directly suppress tumor growth in addition to its effect on EBV suppression.

The Aryl hydrocarbon receptor (AHR) is a ligand-inducible transcription factor that mediates the toxic and carcinogenic effects of xenobiotic or environmental contaminants. It belongs to a member of the basic helix-loop-helix (bHLH)-PER/ARNT/SIM (PAS) family and forms heterodimer with Aryl hydrocarbon receptor nuclear translocator (ARNT) as a co-activator or co-repressor [16]. The bHLH/PAS proteins, along with another member named Hypoxia-inducible factor-1α (HIF1α) [17, 18], are involved in diverse biological processes, including maintaining homeostasis, regulating circadian rhythms, organ development, carcinogenesis and stress response to hypoxia [17, 19–21]. Increased AHR expression may interact with and sequester ARNT, minimizing the ability of ARNT to interact with stabilized HIF1α to induce VEGF production and subsequently suppress the HIF1α pathway [22]. Under normoxic conditions, HIF1α is hydroxylated at specific proline residues (P402, P564) by prolyl hydroxylases, which leads to the rapid degradation of HIF1α proteins through ubiquitinylation and proteasome-mediated proteolysis [23, 24]. Double mutations of P402(A)/P564(A) prevent HIF1α degradation and exert constitutive HIF1α stabilization effects [25].

Cancer cells have been shown to have increased reactive oxygen species (ROS) compared to normal counterparts. This is partly due to an enhanced metabolism and mitochondrial dysfunction in cancer cells [26]. Manganese superoxide dismutase (SOD2) is an antioxidant enzyme located in the mitochondria that can scavenge superoxide anions (O_2_^.-^) to hydrogen peroxide. It has been reported that SOD2 suppression leads to ROS over-generation and subsequently inhibits virus infection [27, 28]. ROS modulates activities of several proteins or signaling pathways involved in tumor cell proliferation, while ROS over-generation can also suppress tumor growth through apoptosis, DNA damage and autophagy [28].

In an effort to develop an efficient strategy for AML treatment, we found that BA could slightly suppress tumor growth through ROS generation and AHR activation with subsequent HIF1α suppression in THP1 cells. We also found that chidamide (CDM) treatment results in ROS generation and increases p300 acetylation, subsequently suppressing HIF1α transcriptional activity. Furthermore, BA/CDM combination additively increases ROS generation with subsequent DNA damage and apoptosis, and significantly suppresses the HIF1α pathway with decreased VEGF expression, subsequently suppressing AML tumor growth from both the *in vitro* and *in vivo* mice model. Overexpression of SOD2 and a constitutive HIF1α (HIF1C) completely reverses the suppression effect of BA/CDM. We conclude that combination of BA/CDM additively inhibits AML through ROS over-generation and HIF1α pathway suppression.

## RESULTS

### Betulinic acid (BA) increases AHR expression by demethylation on the AHR promoter in acute myeloid leukemia cells

**Figure 1:**
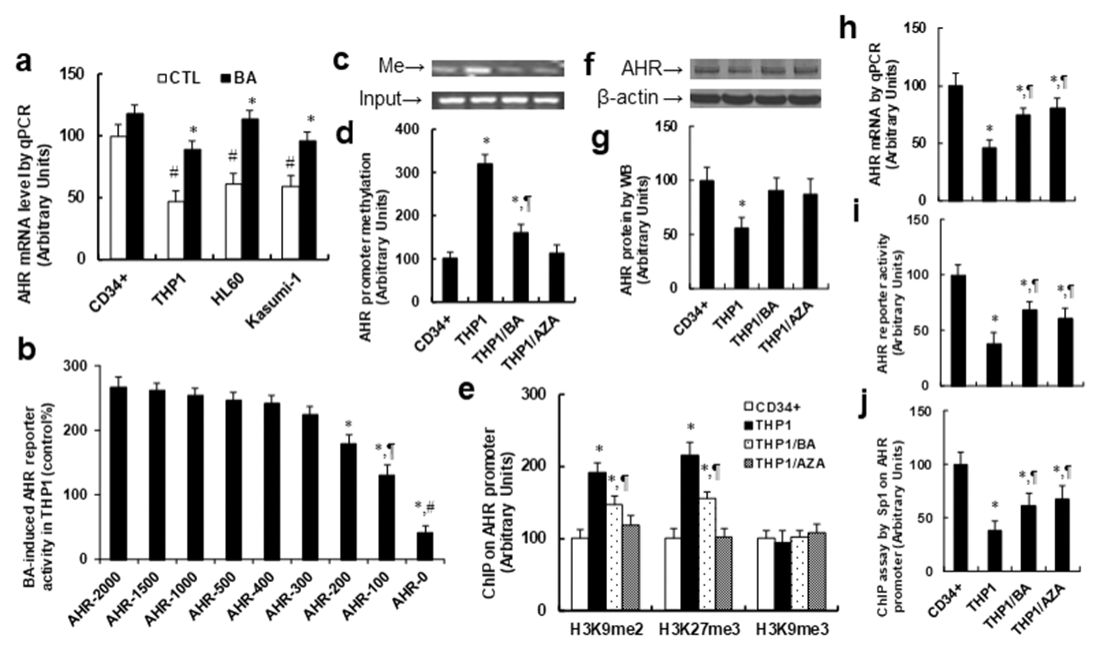
Betulinic acid (BA) increases AHR expression by demethylation on the AHR promoter in acute myeloid leukemia cells **(a)** Different AML cell lines and primary CD34+ were treated by either control (CTL) or BA (5μg/ml betulinic acid) for 24 hours and the mRNA for AHR was measured, n=4. ^*^, *P*<0.05, vs CTL group; #, *P*<0.05, vs CTL in CD34+ group. **(b)** The THP1 cells were transfected with indicated AHR reporter constructs, then treated by either CTL or 15μg/ml BA for 24 hours, the AHR reporter activity was measured, n=5. ^*^, *P*<0.05, vs AHR-2000 group; ¶, *P*<0.05, vs AHR-200 group; #, *P*<0.05, vs AHR-100 group. **(c-j)** The CD34+ or THP1 cells were treated by either BA or AZA (3μM) for 24 hours, and the cells were harvested for further analysis. (c) Representative picture for DNA methylation on AHR promoter. (d) Quantitation of (c), n=5. (e) ChIP analysis on AHR promoter, n=4. (f) Representative picture for AHR proteins. (g) Quantitation of (f), n=5. (h) AHR mRNA by qPCR, n=4. (i) AHR reporter activity, n=5. (j) ChIP analysis by Sp1 antibody on AHR promoter, n=4. ^*^, *P*<0.05, vs CD34+ group; ¶, *P*<0.05, vs THP1 group. Results are expressed as mean ± SEM.

Our preliminary results showed that betulinic acid (BA) suppresses HIF1α transcriptional activity, has no effect on the expression of HIF1α and ARNT, and increases AHR expression. We suppose that BA may suppress HIF1α activity through AHR activation. We first measured the effect of BA on the AHR expression in different acute myeloid leukemia (AML) cell lines, and the primary CD34 positive hematopoietic stem cells (CD34+) were used as a control. In Figure 1a, we found that BA significantly increased the AHR gene expression in AML cell lines, including THP1, HL60 and Kasumi-1, while there was no effect on CD34+ cells. On the other hand, the above 3 AML cell lines have much less basal expression of AHR than primary CD34+ cells. Our results indicate that decreased AHR expression is a common phenomenon in AML cells compared to primary CD34+ cells and BA treatment can restore this effect. We then investigated the mechanisms of BA-mediated AHR activation, and the THP1 cells were selected as the representative of AML cell line for the subsequent experiments. To localize the regulatory elements required for transcriptional activation of AHR gene by BA treatment, progressive 5’ promoter deletion constructs were generated containing different portions of the human AHR promoter. As shown in Figure 1b, the reporter activities were not markedly changed among the -2000, -1500, -1000, -500, -400 and -300 deletion constructs (numbered according to Ensembl Transcript ID: AHR-201 ENST00000242057.8, transcription start site was marked as 0). However, a significant decrease of activity was observed in the -200, -100 and -0 constructs compared to the AHR-2000 control group. These data indicate that elements between -300 and -0 from TSS (transcription start site) on the AHR promoter are responsible for BA-induced transcriptional activation. We then measured the DNA methylation in the location of -300 ∼ 0 on the AHR promoter as indicated previously [29]. In Figure 1c and 1d, THP1 cells showed significantly increased DNA methylation compared to primary CD34+ cells, while this effect was significantly decreased by BA treatment, and was completely diminished by DNA demethylating agent AZA (5-aza-2’-deoxycitidine), indicating that the effect of BA is involved with DNA demethylation. We also measured the epigenetic changes of histone methylation on the AHR promoter using ChIP techniques as shown in Figure 1e. We found that THP1 cells showed significantly increased H3K9 di-methylation (H3K9me2) and H3K27 tri-methylation (H3K27me3) on the AHR promoter compared to primary CD34+ cells, while H3K9 tri-methylation (H3K9me3) did not change. Also, BA treatment significantly decreased, and AZA completely blocked DNA methylation in THP1 cells, indicating that BA-induced AHR activation may be due to BA-mediated DNA demethylation on the AHR promoter. We then measured the effect of BA on AHR activation, and found that THP1 has much lower protein levels (see Figure 1f and 1g), mRNA levels (see Figure 1h) and AHR luciferase reporter activity (see Figure 1i) compared to CD34+ cells, while BA or AZA treatment significantly increased AHR activation in THP1 cells. It has been reported that AHR expression can be suppressed by promoter hypermethylation and subsequently inhibits Sp1 binding to the AHR promoter in human leukemia [29]. We suppose that hypermethylation on the AHR promoter in THP1 cells may inhibit the binding of Sp1 to the AHR promoter. In Figure 1j, we measured the binding ability of Sp1 on the AHR promoter using ChIP techniques. The results showed that THP1 has significantly less binding ability compared to CD34+ cells, while either BA or AZA treatment significantly decreased this effect, indicating that BA-induced AHR activation may be due to BA-mediated DNA demethylation and the subsequent increased Sp1 binding on the AHR promoter.

### Betulinic acid-mediated AHR activation suppresses the HIF1α pathway in THP1 cells

**Figure 2:**
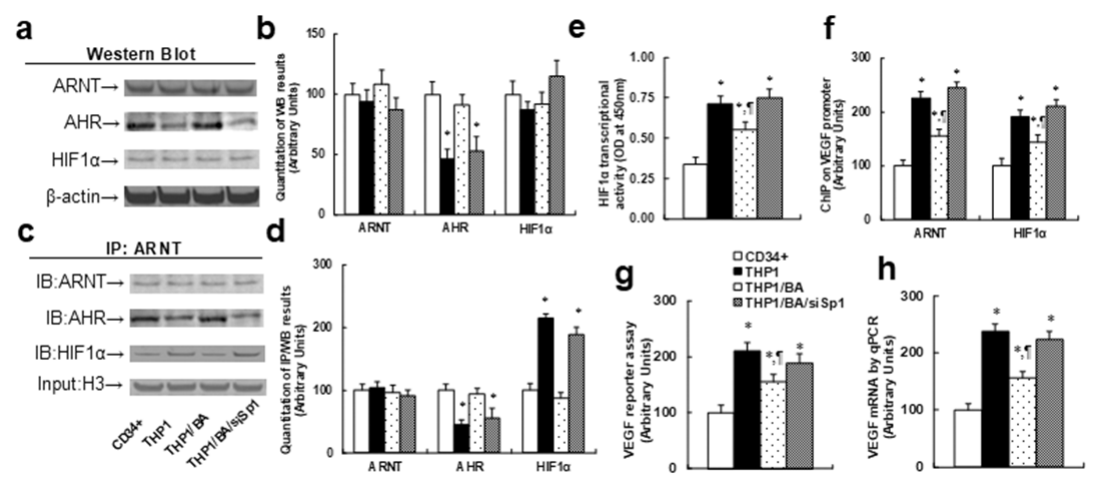
Betulinic acid-mediated AHR expression suppresses the HIF1α pathway in THP1 cells The CD34+ cells or THP1 cells were treated with either control (THP1), THP1/BA (5μg/ml), or BA treatment plus Sp1 knockdown (THP1/BA/siSp1) for 24 hours in hypoxic conditions, and then cells were harvested for further analysis. **(a)** Representative pictures for Western Blotting analysis from nuclear extracts. **(b)** Quantitation of (a), n=5. **(c)** Representative pictures for IP/WB analysis from nuclear extracts, 10% H3 as input control. **(d)** Quantitation of (c), n=5. **(e)** HIF1α transcriptional activity assay, n=5. **(f)** ChIP analysis on VEGF promoter, n=4. **(g)** VEGF luciferase reporter assay, n=5. **(h)** VEGF mRNA level by qPCR, n=4. ^*^, *P*<0.05, vs CD34+ group; ¶, *P*<0.05, vs THP1 group. Results are expressed as mean ± SEM.

We measured the effect of BA-mediated AHR activation on the HIF1α pathway. In Figure 2a and 2b, the western blotting results showed that the protein levels of ARNT and HIF1α were not significantly different in treated cells under hypoxic conditions, while AHR protein levels in THP1 cells were 50% of that of CD34+ cells. This effect was completely restored by BA treatment, and Sp1 knockdown (siSp1) diminished the effect of BA. In Figure 2c and 2d, the immunoprecipitation (IP) results showed that AHR has decreased and HIF1α has increased association with ARNT in THP1 cells compared to CD34+ cells, while BA treatment completely restored this effect, and siSp1 treatment mimicked this effect. Our results indicate that AHR and HIF1α are competitively binding with ARNT, and decreased AHR expression in THP1 cells has less binding capacity with ARNT, indirectly increasing the binding ability of HIF1α with ARNT and subsequently activating the HIF1α pathway. Next, we measured the effect of BA-mediated AHR activation on the HIF1α target gene VEGF. In Figure 2e, THP1 cells have more than 2 times higher HIF1α transcriptional activity than CD34+ cells, BA treatment partly decreased HIF1α activity, and BA treatment in Sp1 knockdown THP1 cells (THP1/BA/siSp1) completely diminished the BA effect. In Figure 2f, we measured the binding ability of ARNT and HIF1α on the VEGF promoter using ChIP techniques. We found that both ARNT and HIF1α have an increased binding ability to the VEGF promoter in THP1 cells compared to CD34+ cells, BA treatment partly decreased binding in THP1 cells, and in Sp1 knockdown cells (THP1/BA/siSp1), the effect of BA was completely diminished. Lastly, we measured VEGF reporter activity (see Figure 2g) and VEGF mRNA (see Figure 2h). The results showed that THP1 cells have more than 2 times higher reporter activity and mRNA levels than CD34+ cells, while this effect was partly restored by BA treatment, but the BA effect was completely diminished in Sp1 knockdown cells (THP1/BA/siSp1), indicating that BA treatment could suppress the HIF1α pathway in THP1 cells.

### Chidamide (CDM) treatment suppresses the HIF1α pathway in THP1 cells

**Figure 3:**
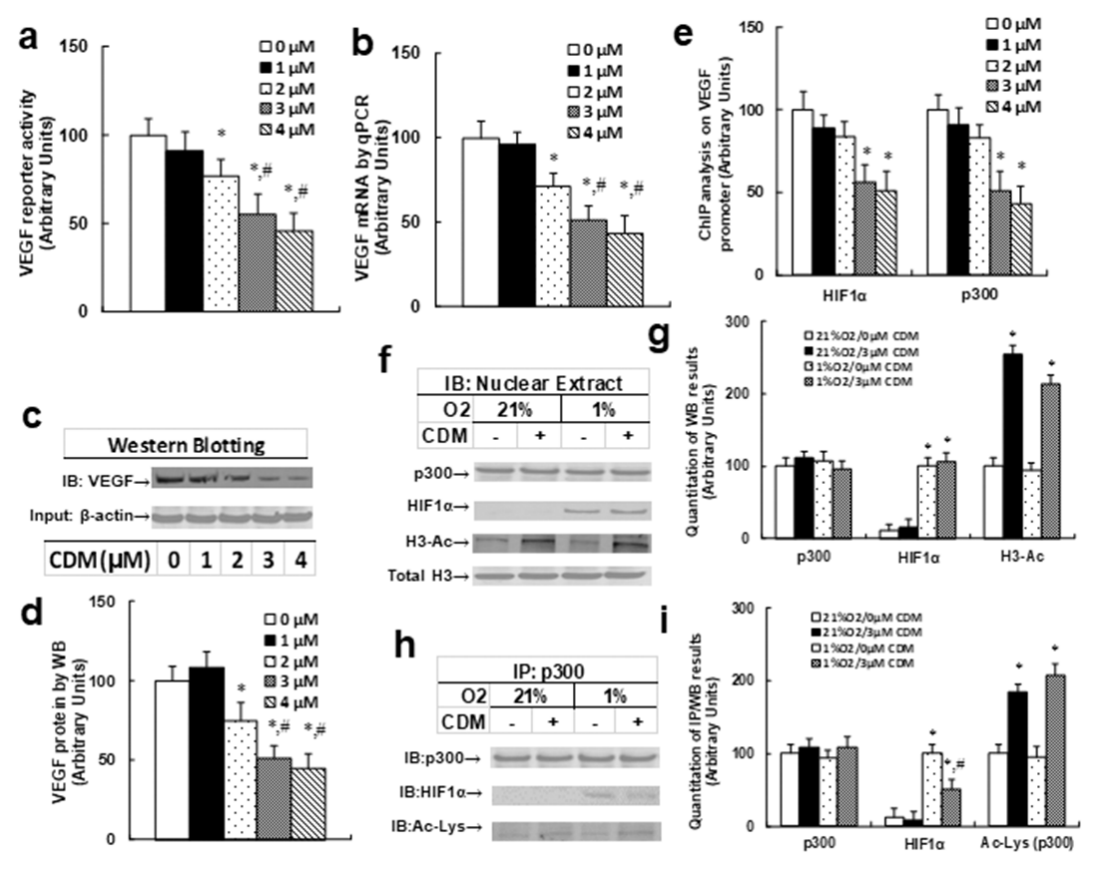
Chidamide (CDM) treatment suppresses the HIF1α pathway in THP1 cells **(a-e)** The THP1 cells were treated with different concentrations of chidamide (CDM) for 24 hours in hypoxic conditions, and the cells were harvested for analysis. (a) VEGF reporter activity, n=5. (b) VEGF mRNA level by qPCR, n=4. (c) Representative picture for VEGF western blots. (d) Quantitation of (c), n=5. (e) ChIP analysis on VEGF promoter, n=4. ^*^, *P*<0.05, vs 0 μM Chidamide; #, *P*<0.05, vs 2 μM Chidamide. **(f-i)** THP1 cells were treated by either 0 or 3μM CDM for 2 days under normoxic (21% O2) or hypoxic (1% O2) conditions, then the cells were harvested for further analysis. (f) Representative pictures for Western Blotting analysis. (g) Quantitation of (f), n=5. (h) Representative pictures for IP/WB analysis. (i) Quantitation of (h), n=5. ^*^, *P*<0.05, vs 21%O2/0μM CDM; #, *P*<0.05, vs 1% O2/3μM CDM. Results are expressed as mean ± SEM.

We first measured the effect of chidamide (CDM) treatment on VEGF expression. Different concentrations of CDM were used to treat THP1 cells for 24 hours according to a previously published article [13], and the VEGF reporter activity (see Figure 3a), VEGF mRNA (see Figure 3b), and VEGF protein levels (see Figure 3c and 3d) were measured. We found that 1μM CDM showed little effect compared to 0μM CDM, while 2μM CDM significantly decreased VEGF reporter activity, mRNA and protein levels, and 3μM and 4μM CDM further decreased VEGF expression compared to 2μM CDM. We then measured the effect of 3μM CDM on the association of p300 and HIF1α under either normoxic or hypoxic conditions. In Figure 3f and 3g, we found that the p300 protein level did not change under different treatments; HIFα expression is not detectable under normoxic conditions, but is detectable under hypoxic conditions, but there is no effect with CDM treatment. Furthermore, we found that histone 3 acetylation (H3-Ac) was significantly increased with CDM treatment, but there was no difference between normoxic and hypoxic conditions. Our results showed that CDM treatment increased histone 3 acetylation but did not change the protein expression of p300 and HIF1α. In Figure 3h and 3i, we measured the p300 acetylation and interaction with HIF1α using IP/WB techniques. Our results showed that p300 had increased acetylation with CDM treatment under both normoxic and hypoxic conditions. Furthermore, p300 showed decreased association with HIF1α under hypoxic conditions. Our results indicate that CDM-mediated p300 over-acetylation suppressed the interaction of p300 with HIF1α, and subsequently suppressed the HIF1α pathway with decreased expression of HIF1α target gene VEGF.

### Combination of betulinic acid (BA) and chidamide (CDM) additively potentiates ROS formation and related cell damage, while SOD2 overexpression diminishes this effect

**Figure 4:**
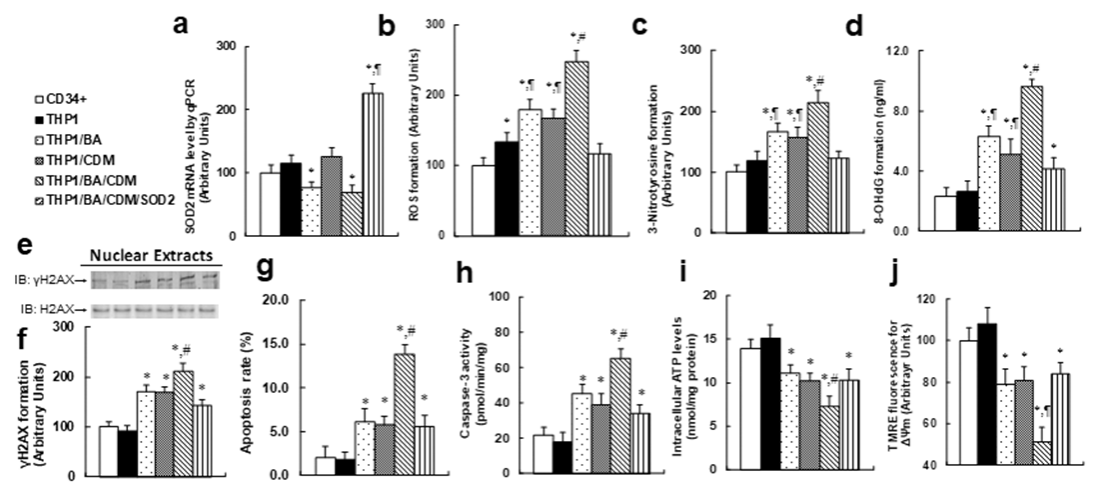
Combination of betulinic acid (BA) and chidamide (CDM) additively potentiates ROS formation and related cell damage, while SOD2 overexpression diminishes this effect The CD34+ or THP1 cells were treated with either control, 15μg/ml BA alone (BA), 3μM CDM alone (CDM), combination of BA and CDM (BA/CDM), or BA/CDM combination in SOD2 overexpression cells (THP1/BA/CDM/SOD2) for 24 hours in hypoxic conditions, and the cells were harvested for further analysis. **(a)** SOD2 mRNA level by qPCR, n=4. **(b)** ROS formation, n=5. **(c)** 3-Nitrotyrosine formation, n=5. **(d)** 8-OHdG formation, n=5. **(e)** Representative pictures for γH2AX formation. **(f)** Quantitation of (e), n=5. **(g)** Apoptosis rate by TUNEL assay, n=5. **(h)** Caspase-3 activity, n=5. **(i)** Intracellular ATP level, n=5. **(j)** ∆ᴪm by TMRE fluorescence, n=5. ^*^, *P*<0.05, vs CD34+ group; ¶, *P*<0.05, vs THP1 group; #, *P*<0.05, vs THP1/BA group. Results are expressed as mean ± SEM.

We measured the combination effect of BA and CDM on ROS generation and the subsequent cell damage. The CD34+ or THP1 cells were treated with either control, BA alone, CDM alone, BA/CDM combination, or BA/CDM combination in SOD2 overexpression cells (THP1/BA/CDM/SOD2) for 24 hours in hypoxic conditions, and cells were harvested for further analysis. We first measured the SOD2 expression (see Figure 4a), and the results showed that BA or BA/CDM treatment somewhat decreased SOD2 expression but was statistically significant, CDM alone showed no effect, while BA/CDM/SOD2 significantly increased SOD2 expression, indicating a successful lentivirus-mediated SOD2 manipulation in THP1 cells. We then measured oxidative stress, including ROS formation (see Figure 4b) and 3-nitrotyrosine (see Figure 4c) formation, and DNA damage, including 8-OHdG (see Figure 4d) and γH2AX formation (see Figure 4e and 4f). It showed that either BA or CDM alone increased oxidative stress and DNA damage, BA/CDM combination further potentiated the effect, and SOD2 overexpression significantly diminished this effect in THP1 cells. On the other hand, there was no significant difference between primary CD34+ and THP1 cells, except that THP1 cells has slightly higher ROS formation than CD34+ cells in the absence of any treatment. We then measured the apoptosis rate and caspase-3 activity from the above treated cells. The results showed that the apoptosis rate (see Figure 4g) and caspase-3 activity (see Figure 4h) were slightly increased with either BA or CDM treatment alone, while BA/CDM combination treatment significantly potentiated this effect, indicating an additive effect of BA and CDM. We also measured the intracellular ATP level (see Figure 4i) and mitochondrial membrane potential (∆ᴪm) using TMRE fluorescence (see Figure 4j), and found that both factors were decreased with either BA or CDM alone, while BA/CDM combination additively potentiated this effect. On the other hand, SOD2 overexpression partly diminished the combination effect of BA/CDM, indicating that BA/CDM-mediated ROS formation plays an important role in BA/CDM-mediated tumor suppression.

### Combination of betulinic acid (BA) and chidamide (CDM) additively suppresses the HIF1α pathway, and overexpression of constitutive HIF1α (HIF1C) diminishes this effect

**Figure 5:**
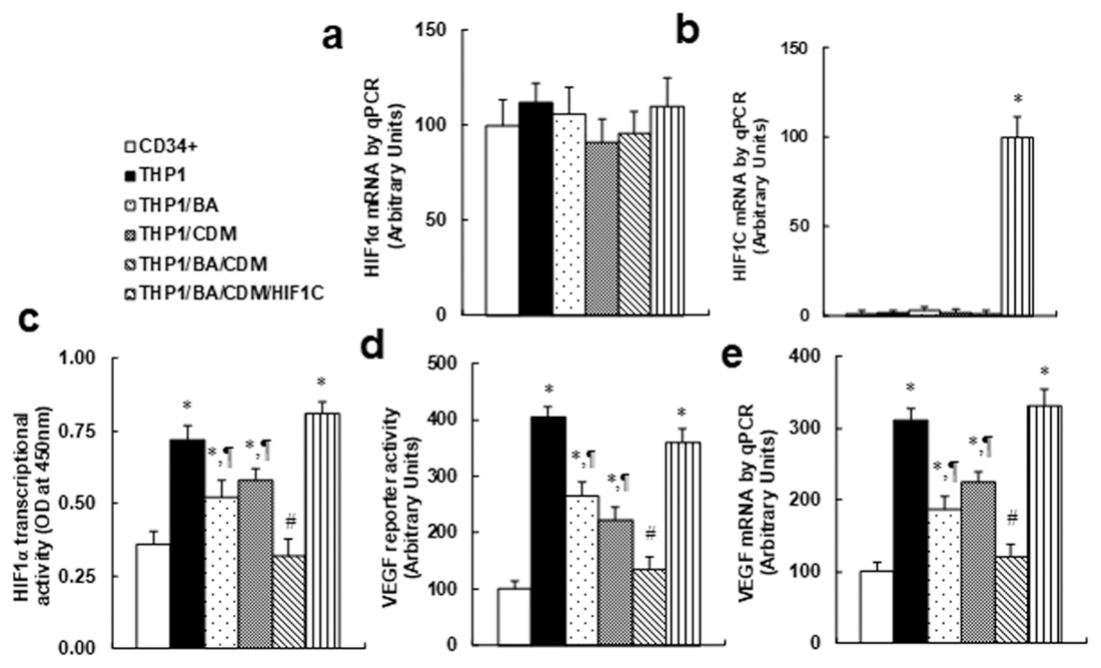
Combination of betulinic acid (BA) and chidamide (CDM) additively suppresses the HIF1α pathway, and overexpression of constitutive HIF1α (HIF1C) diminishes this effect The CD34+ or THP1 cells were treated with either control, 15μg/ml BA alone (BA), 3μM CDM alone (CDM), BA/CDM combination, or BA/CDM combination in HIF1C overexpression cells (THP1/BA/CDM/HIF1C) for 24 hours in hypoxic conditions, and the cells were harvested for further analysis. **(a-b)** Specific primers were designed to measure mRNA levels for endogenous HIF1α (a) and constitutive HIF1α (HIF1C) (b), n=4. **(c)** HIF1α transcriptional activity assay, n=5. **(d)** VEGF reporter activity, n=5. **(e)** VEGF mRNA level by qPCR, n=4. ^*^, *P*<0.05, vs CD34+ group; ¶, *P*<0.05, vs THP1 group; #, *P*<0.05, vs THP1/BA group. Results are expressed as mean ± SEM.

We measured the combination effect of BA/CDM on the HIF1α pathway. The CD34+ or THP1 cells were treated with either control, BA alone, CDM alone, BA/CDM combination, or BA/CDM combination in HIF1C overexpression cells (THP1/BA/CDM/HIF1C) for 24 hours in hypoxic conditions. We first measured the mRNA levels for endogenous HIF1α and constitutive HIF1α (HIF1C) using specific primers as shown in Table 1. In Figure 5a, endogenous HIF1α showed no significant difference in different treatments, while in Figure 5b, the constitutive HIF1α (HIF1C) was not detectable in any of the treatments except the THP1/BA/CDM/HIF1C group, indicating a successful and sufficient HIF1C manipulation using lentivirus-carrying HIF1C infection. In Figure 5c, we measured the HIF1α transcriptional activity and found that THP1 cells have significantly higher HIF1α activity than primary CD34+ cells, and this activity was significantly decreased by either BA or CDM treatment alone, and BA/CDM combination decreased activity further compared to the BA or CDM group, while overexpression of HIF1C (constitutive HIF1α) completely diminished this effect. Further investigation showed that both VEGF reporter activity (see Figure 5d) and VEGF mRNA (see Figure 5e) were significantly higher in THP1 cells compared to CD34+ cells, while this effect was decreased by either BA or CDM treatment alone, and was further decreased in BA/CDM combination, but again, it was completely diminished by HIF1C overexpression. Our results indicate that combination of BA/CDM additively suppresses the HIF1α pathway, and a constitutive HIF1α (HIF1C) diminishes this effect.

**Table 1:** Sequences of primers for the real time quantitative PCR (qPCR)

Gene	Species	Analysis	Forward primer (5’→3’)	Reverse primer (5’→3’)
β-actin	Human	mRNA	gatgcagaaggagatcactgc	atactcctgcttgctgatcca
SOD2	Human	mRNA	gcctacgtgaacaacctgaac	tgaggtttgtccagaaaatgc
AHR	Human	mRNA	gttgtgatgccaaaggaagaa	tcatgccactttctccagtct
VEGF	Human	mRNA	gccagcacataggagagatga	catttacacgtctgcggatct
HIF1α	Human	mRNA	tttgctggccccagccgct	tctgtaatttttcgttggg
HIF1C	Human	mRNA	tttgctggccgcagccgct	tctgtaatttttcgttggg
AHR	Human	ChIP	aagttagctgacccaccgtct	cgtgatgacgtaggacgtaag
VEGF	HUman	ChIP	gggagccagagaccagtg	cccaaaacttttcccaaactc

### Combination of betulinic acid (BA) and chidamide (CDM) additively suppresses tumor cell growth, and overexpression of SOD2 and constitutive HIF1α (HIF1C) diminishes this effect

**Figure 6:**
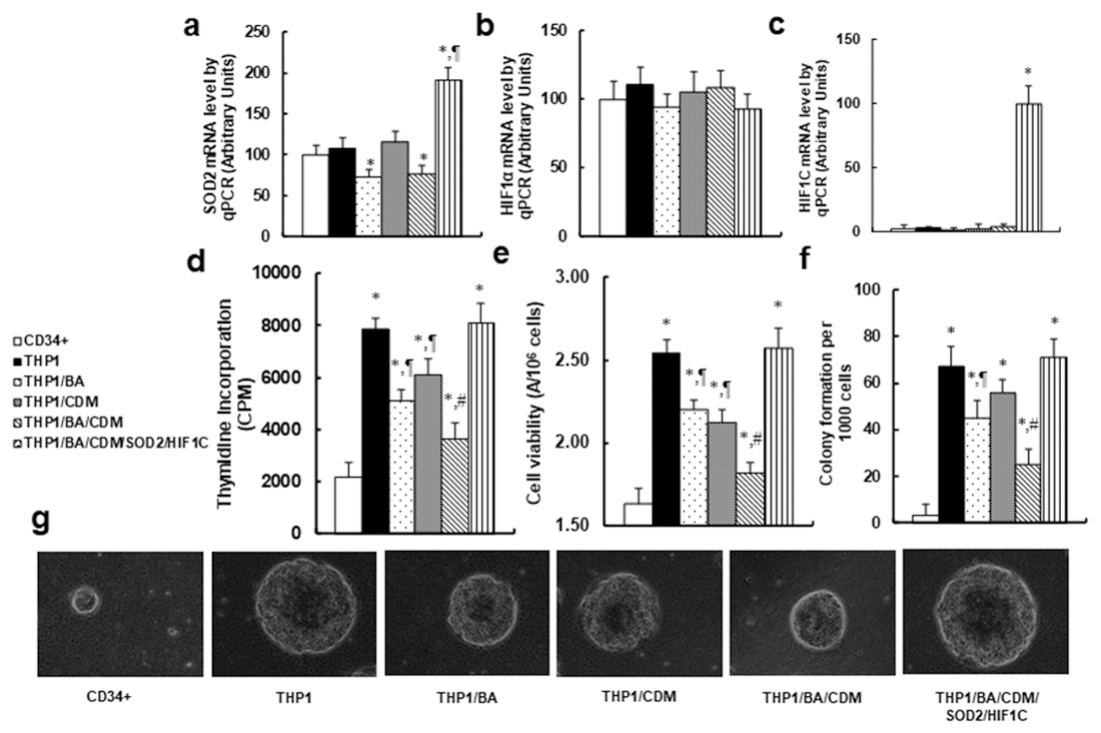
Combination of betulinic acid (BA) and chidamide (CDM) additively suppresses tumor cell growth; overexpression of SOD2 and constitutive HIF1α (HIF1C) diminishes this effect The CD34+ or THP1 cells were treated with either control, 15μg/ml BA alone (BA), 3μM CDM alone (CDM), BA/CDM combination, or BA/CDM combination in SOD2 and HIF1C overexpression cells (THP1/BA/CDM/SOD2/HIF1C) for 24 hours in hypoxic conditions, and the cells were harvested for further analysis. **(a-c)** mRNA levels for SOD2 (a), endogenous HIF1α (b) and constitutive HIF1α (HIF1C) (c), n=4. **(d)** The cell proliferation analysis by thymidine incorporation, n=5. **(e)** Cell viability analysis by MTT assay, n=5. **(f)** Colony formation assay in soft agar, n=5. **(g)** Representative picture for colony formation (f). ^*^, *P*<0.05, vs CD34+ group; ¶, *P*<0.05, vs THP1 group; #, *P*<0.05, vs THP1/BA group. Results are expressed as mean ± SEM.

We measured the combination effect of BA/CDM on tumor cell growth through ROS formation and HIF1α pathway suppression. The CD34+ or THP1 cells were treated by either control, BA alone, CDM alone, BA/CDM combination, or BA/CDM combination in SOD2/HIF1C overexpression cells (THP1/BA/CDM/SOD2/HIF1C) for 24 hours in hypoxic conditions, and the cells were harvested for further analysis. We first measured the mRNA levels for SOD2, endogenous HIF1α and constitutive HIF1α (HIF1C) using specific primers as shown in Table 1. In Figure 6a, SOD2 expression was significantly decreased in THP1/BA and THP1/BA/CDM treatments, while it was increased by around twofold in THP1/BA/CDM/SOD2/HIF1C group, indicating that the manipulation of SOD2 expression using SOD2 lentivirus infection was sufficient. In Figure 6b, endogenous HIF1α showed no significant difference in different treatments, while in Figure 6c, the constitutive HIF1α (HIF1C) was not detectable in any of the treatments except the THP1/BA/CDM/SOD2/HIF1C group. Our results indicate a successful and sufficient HIF1C manipulation using lentivirus-carrying HIF1C infection. We then measured the cell proliferation using thymidine incorporation (see Figure 6d) and cell viability using the MTT assay (see Figure 6e). We found that both cell proliferation and viability were significantly increased in THP1 cells compared to CD34+ cells, and this effect was decreased in either BA alone or CDM alone, and was decreased further in BA/CDM combination, while this effect was completely reversed by overexpression of SOD2 and constitutive HIF1α. We also measured the *in vitro* colony formation in soft agar (see Figure 6f and 6g). Our results showed a pattern of effects similar to that of cell proliferation and viability. BA/CDM combination further decreased colony formation compared to BA or CDM alone, and SDO2/HIF1C overexpression completely diminished this effect. Our results indicate that BA/CDM combination suppresses tumor growth through ROS formation and HIF1α pathway suppression.

### Combination of betulinic acid (BA) and chidamide (CDM) additively potentiates oxidative stress and suppresses tumor growth in *in vivo* xenograft tumor development, while overexpression of SOD2 and constitutive HIF1α (HIF1C) diminishes this effect

**Figure 7:**
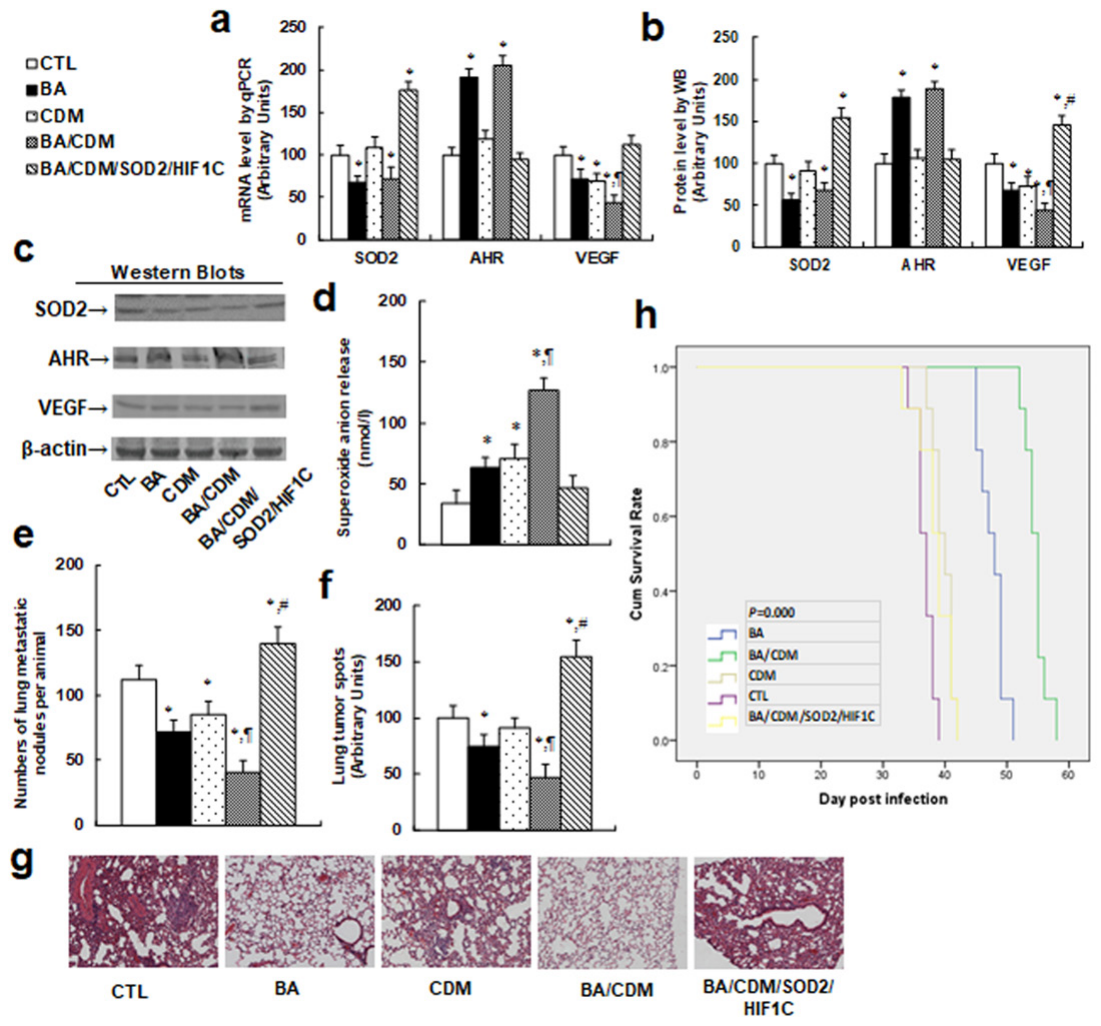
Combination of betulinic acid (BA) and chidamide (CDM) additively potentiates oxidative stress and suppresses tumor growth in *in vivo* xenograft tumor development, while overexpression of SOD2 and constitutive HIF1α (HIF1C) diminishes this effect The nude mice were injected through the tail vein for *in vivo* xenograft tumor development study, then treated with BA or CDM alone, a BA/CDM combination, or a BA/CDM combination with SOD2 and HIF1C overexpressed THP1 cells (BA/CDM/SOD2/HIF1C), and then the treated mice were sacrificed for further analysis. **(a)** mRNA level by qPCR, n=4. **(b)** Quantitation of protein levels by western blots, n=5. **(c)** Representative pictures for (b). **(d)** Superoxide anion release from tumor tissues, n=5. **(e)** Tumor colony formation in lung, n=5. **(f)** Mice were killed upon 20% weight loss, and the lung were harvested for terminal analysis. The metastatic tumor nodules from the lungs were counted, and then the formalin-fixed, paraffin-embedded tumor tissue from the lung were sectioned to 4mm thickness, and the histopathological analyses were performed with H&E staining. Images were taken using a Carl Zeiss MIRAX MIDI slide scanner, and the lung tumor spots were analyzed using a 3DHISTECH Pannoramic Viewer, n=5. **(g)** Representative picture by H&E staining for (f). **(h)** Kaplan-Meier analysis comparing survival of mice between each treatment group, *P* value represents log-rank Mantel-Cox test result, n=9. ^*^, *P*<0.05, vs CTL group; ¶, *P*<0.05, vs BA group; #, *P*<0.05, vs BA/CDM group. Results are expressed as mean ± SEM.

We evaluated the combination effect of BA/CDM on tumor suppression through an *in vivo* xenograft tumor development study using THP1 cells, and we also investigated the potential role of SOD2 and HIF1α through lentivirus-carrying SOD2/HIF1C overexpression in THP1 cells. In Figure 6, the nude mice were injected through the tail vein with THP1 cells or SOD2/HIF1C overexpression THP1 cells. The mice were then treated with either BA or CDM alone, or BA/CDM combination. The subsequent xenograft tumor tissues from the lungs were isolated and analyzed, and mouse survival was calculated. We first measured the gene expression from tumor tissues, including SOD2, AHR and VEGF, for both mRNA (see Figure 7a) and protein levels (see Figure 7b and 7c). The results showed that either BA alone, or BA/CDM combination, decreased SOD2 expression but increased AHR expression, while CDM alone had no effect on the expression of SOD2 and AHR. On the other hand, either BA or CDM treatment alone decreased VEGF expression, and BA/CDM combination additively suppressed VEGF expression, while expression of SOD2/HIF1C completely reversed VEGF suppression, indicating that lentivirus-carrying SOD2/HIF1C manipulation in THP1 cells and *in vivo* chemical treatment work efficiently. We then measured the superoxide anion (O_2_^-.^) release from the xenograft tumor tissues. In Figure 7d, BA or CDM alone slightly increased superoxide anion release, and the BA/CDM combination additively increased superoxide anion release by more than 3 times compared to the control (CTL) group. Overexpression of SOD2/HIF1C completely diminished BA/CDM-mediated O_2_^-.^ release. We then measured the lung tumor nodules formation (see Figure 7e) and lung tumor spots by H&E staining (see Figure 7f and 7g). We found that BA or CDM alone slightly decreased tumor formation, BA/CDM combination additively suppressed tumor formation, SOD2/HIF1C expression (BA/CDM/SOD2/HIF1C) completely diminished the inhibition effect of BA/CDM and largely potentiated tumor growth compared to the CTL group. We finally measured the mouse survival rate using Kaplan-Meier analysis (see Figure 7h). The results showed that CDM alone slightly increased mouse survival, BA significantly increased it, and BA/CDM combination additively further increased mouse survival. On the other hand, SOD2/HIF1C overexpression (BA/CDM/SOD2/HIF1C) completely diminished the effect of BA/CDM and significantly decreased mouse survival rate compared to the control (CTL) group. Our results showed that BA/CDM combination additively suppresses *in vivo* tumor growth in THP1 cells, and overexpression of SOD2/HIF1C diminishes this inhibition effect, indicating that BA/CDM exert additive inhibition effect on tumor growth through ROS generation and HIF1α pathway suppression.

## DISCUSSION

In this study, we demonstrate that betulinic acid (BA) suppresses AML cells through SOD2 suppression with subsequent ROS generation and AHR activation with subsequent HIF1α suppression; Chidamide (CDM) slightly suppresses AML cells through ROS generation and increased p300 acetylation with subsequent dissociation of p300 with HIF1α. Combination of BA and CDM achieve an additive suppression effect on AML cells through ROS over-generation and HIF1α suppression with decreased VEGF expression. This is the first time we have shown the potential effect and possible mechanism of BA and CDM on the inhibition of AML cells through ROS generation and HIF1α suppression.

### Betulinic acid-mediated selective cytotoxicity to tumor cells

We have found that BA could achieve a selective cytotoxic effect on AML tumor cells, instead of primary CD34+ cells [30], which shows potential application for anti-tumor drug development. Even so, the cytotoxicity *in vitro* could not accurately reflect the real toxicity of BA in the human body, but very little cytotoxicity to the normal cells should be considered very carefully in anti-tumor drug development. Tumor cells are distinguished from normal cells by their characteristics of being able to survive with lower levels of or even without oxygen (in anaerobic situations) while generating much more ATP and consuming much more glucose to lactic acid (low pH) than normal cells through glycolysis. Generally, tumors have the following factors that could distinguish them from normal cells, which include (a) increased glycolysis in anaerobic situations, (b) diminished apoptosis procedure, (c) interrupted electron transport chain (ETC) and mitochondrial function, and (d) increased glucose consuming and ATP generation. Here from our work, we found that BA could trigger several of the following effects that are specific to tumor cells: (a) BA could inhibit HIF1α and its downstream target gene VEGF, which may increase angiogenesis in the anaerobic condition [7]. (b) BA interrupts the glycolysis procedure as reflected by decreased lactate release, which result in decreased ATP generation and MMP that lead to apoptosis [31, 32]. (c) The strong redox effect of BA initiates a large increase of mitochondrial ROS generation that may further induce mitochondrial dysfunction and apoptosis, especially in ETC-interrupted tumor cells [33].

### Betulinic acid-mediated AHR activation and HIF1α pathway suppression

BA treatment significantly increases the AHR expression in AML cells, while it has no effect on CD34+ cells. Further investigation shows that BA treatment demethylates the AHR promoter in THP1 cells, and subsequently activates AHR expression through increased Sp1 binding ability on the AHR promoter. Specificity protein 1 (Sp1) is a well-identified transcription factor that recognizes GC-box and interacts with DNA on related promoters to regulate gene expression. We show that Sp1 knockdown (siSp1) suppresses AHR expression and subsequently activates the HIF1α pathway with increased VEGF expression in THP1 cells. On the other hand, Sp1 has been reported to be overexpressed in some tumors [7, 34], indicating that Sp1 may play an important but different role in tumor development through regulation of down-stream target genes in different cell types. Furthermore, we show that BA treatment increases AHR expression in THP1 cells and then competitively binds with ARNT, and subsequently decreases the association of ARNT with HIF1α, indirectly suppressing the HIF1α pathway with decreased VEGF expression. In this study, we show thatAML cells have lower AHR expression compared to primary CD34+ cells, and BA treatment increases AHR expression in AML cells and subsequently suppresses the HIF1α pathway with decreased VEGF expression. It seems that AHR activation in AML cells plays an inhibition effect on AML tumor growth [29]; this is consistent with the recent finding that AHR may promote HIF1α degradation in lymphocyte metabolism and indirectly suppress the HIF1α pathway [35]. It has been reported that AHR plays an important endogenous role in hepatic homeostasis. AHR null mice showed small liver sizes with portal fibrosis and early lipid accumulation [19, 36], and AHR plays a positive regulator in cell proliferation [20, 37]. Also, it interacts with signaling pathways controlling cell adhesion and migration [38, 39], indicating that AHR expression may activate hepatocellular carcinoma development, which is different from our findings. Therefore, AHR expression may play an either positive or negative role in tumor development based on different cell types [40, 41].

### Chidamide-mediated HIF1α pathway suppression

HDAC inhibitors (HDACi) have been widely used for new therapeutics of cancers since they can repress tumor growth and angiogenesis [42, 43]. HDACi can enhance the transactivation activity of a variety of transcription factors, but represses HIF1α [44]. Also, the p300 can interact with and activate HIF1α, and over-acetylation of p300 may suppress HIF1α activity [45]. It has been reported that the new type of HDACi chidamide (CDM) can achieve a complete remission in a relapsed and refractory AML patient with MLL-AF9 translocation [14], indicating that CDM may be a good candidate for AML treatment. The THP-1 cell line was generated from an AML sample harboring the MLL-AF9 translocation. In our study, we found that CDM can significantly increase p300 acetylation and suppress association of p300 with HIF1α, subsequently suppressing the HIF1α pathway with decreased VEGF expression in the THP1 cell line. Our results are consistent with the previous report [14], and further proves that chidamide may have a potential suppression effect on AML tumors. Chidamide-mediated p300 over-acetylation and the subsequent HIF1α pathway suppression may provide a new sight for anti-leukemia drug development.

Taken together, we demonstrate that betulinic acid (BA) treatment increases AHR activation with subsequent HIF1α suppression together with decreased SOD2 expression with ROS generation in AML cells. Chidamide (CDM) treatment increases p300 over-acetylation with subsequent HIF1α suppression together with ROS generation in AML cells. Combination of AB/CDM exerts additive inhibition effect on AML tumor growth through HIF1α pathway suppression and ROS over-generation. We conclude that BA/CDM combination could be a sufficient new strategy in developing anti-tumor therapy for AML patients.

## MATERIALS AND METHODS

The human AML cell lines Kasumi-1, HL-60 and THP-1 were obtained from ATCC and maintained in RPMI-1640 media supplemented with 2mM L-glutamine, 10% FBS and standard antibiotics (Lonza). The CD34 positive hematopoietic stem cells (CD34+ HSCs) were isolated from fresh whole blood (obtained from healthy donors) using the EasySepTM Whole Blood CD34 Selection Kit (#18086, STEMCELL Technologies). The purity of CD34+ cells ranging between 93% and 97% was determined by flow cytometry. The cells were first labeled using Anti-Human CD34 Antibody, Clone 581 (#60013, STEMCELL Technologies) followed by Goat Anti-Mouse IgG (H+L) Antibody, Polyclonal, FITC (#60138FI, STEMCELL Technologies). The isolation protocol was approved by the Ethics Committee of Peking University Shenzhen Hospital. The isolated CD34+ HSCs cells were cultured in StemSpan™ serum-free media with the addition of cytokines and supplements (obtained from StemSpan™ CD34+ Expansion Supplement). All cells were maintained in a humidified incubator with 5% CO2 at 37°C. The Hypoxia condition was induced by incubating in 94% N2, 5% CO2 and 1% O2 for 24 hours.

Antibodies for AHR (sc-133088), ARNT (sc-55526) and β-actin (sc-47778), HIF1α (sc-13515), SOD2 (sc-30080), Sp1 (sc-17824) and VEGF (sc-7269) were obtained from Santa Cruz Biotechnology. Antibodies for acetyl-histone H3 (#06-599) and histone H3 (#05-499) were obtained from EMD Millipore. Antibodies for anti-acetyl lysine (ab21623), H2AX (ab20669) and γH2AX (ab2893) were obtained from Abcam, 3-nitrotyrosine (3-NT) was measured by 3-Nitrotyrosine ELISA Kit (ab116691 from Abcam), and the HIF1α transcriptional activity was measured by HIF1α Transcription Factor Assay Kit (ab113104 from Abcam) in 50μl nuclear extracts from treated cells per manufacturers’ instructions. Nuclear extracts were prepared using the NE-PER Nuclear and Cytoplasmic Extraction Reagents Kit (Pierce Biotechnology). The protein concentration was measured using the Coomassie Protein Assay Kit (Pierce Biotechnology) per manufacturers’ instructions. The siRNA for Sp1 (# 4457308) and negative control (# AM4636) were obtained from Ambion, and was transfected by Lipofectamine^®^ 2000 Reagent (Invitrogen). Luciferase activity assay was carried out using the Dual-Luciferase™ Assay System (Promega) and the transfection efficiency was normalized using a cotransfected renilla plasmid.

Chidamide (CDM, CS055) was supplied by Chipscreen Biosciences (Shenzhen, China) and was dissolved in DMF (dimethyl-formamide). For the *in vivo* experiments, CDM was suspended in 0.1% sodium carboxyl methylcellulose and stored at 4°C. Betulinic acid (BA) was purchased from Sigma, and the compounds were dissolved in DMSO (dimethyl sulfoxide) to make a stock solution. The final concentration of the above solvents did not exceed 0.5% in any experiment. The DNA demethylating agent 5-aza-2’-deoxycitidine (AZA, Sigma) was first dissolved by DMSO to achieve 50 mg/ml solution, and then it was further diluted by saline for the final concentration of 3 μM AZA with 24 hours’ treatment.

### Construction of plasmids and vectors

The human genomic DNA was prepared from the above CD34+ HSCs cells. In order to construct the VEGF reporter plasmid, the VEGF gene promoter (Ensembl gene ID: VEGFA-201 ENST00000230480.10) was amplified by PCR and subcloned into the pGL3-basic vector (# E1751, Promega) using restriction sites of Mlu I and Hind III with the following primers: Forward: 5’-gcgc-acgcgt- ctg tga acc ttg gtg ggg gtc -3’ (Mlu I) and Reverse: 5’- gtac- aagctt- ctc gag agg tca cct tcc cgc -3’ (Hind III). In order to construct AHR reporter plasmid, the AHR gene promoter (Ensembl gene ID: AHR-201 ENST00000242057.8) was amplified by PCR and subcloned into pGL3-basic vector using restriction sites of Xho1 and Hind III with the following primers: Forward: 5’- tcga-ctcgag- aag gta agt tca tgt cac tat -3’ (Xho1) and Reverse: 5’- gtac- aagctt- gtt ttc tgc acc ggc ttc cgc -3’ (Hind III). For mapping of AHR promoter activity, the related deletion promoter constructs were generated by PCR methods and subcloned into the pGL3-basic vector. All the vectors were verified by sequencing, and detailed information on these plasmids is available upon request.

### RT reaction and real-time quantitative PCR

Total RNA from treated cells was extracted using the RNeasy Micro Kit (Qiagen), and the RNA was reverse transcribed using an Omniscript RT kit (Qiagen). All the primers were designed using Primer 3 Plus software with the Tm at 60°C, primer size as 21bp, and the product length in the range of 140-160bp (see Table 1). The primers were validated with the amplification efficiency in the range of 1.9-2.1, and the amplified products were confirmed with agarose gel. The real-time quantitative PCR was run on iCycler iQ (Bio-Rad) with the Quantitect SYBR green PCR kit (Qiagen). The PCR was performed by denaturing at 95°C for 8 min, followed by 45 cycles of denaturation at 95°C, annealing at 60°C, and extension at 72°C for 10s, respectively. 1 μl of each cDNA was used to measure target genes. The β-actin was used as the housekeeping gene for transcript normalization, and the mean values were used to calculate relative transcript levels with the ^ΔΔ^CT method per instructions from Qiagen. In brief, the amplified transcripts were quantified by the comparative threshold cycle method using β-actin as a normalizer. Fold changes in gene mRNA expression were calculated as 2^−ΔΔCT^ with CT = threshold cycle, ΔCT=CT (target gene)-CT(β-actin), and the ΔΔCT =ΔCT (experimental)-ΔCT (reference).

### Western blotting

Cells were lysed in an ice-cold lysis buffer (0.137M NaCl, 2mM EDTA, 10% glycerol, 1% NP-40, 20mM Tris base, pH 8.0) with protease inhibitor cocktail (Sigma). The proteins were separated in 10% SDS-PAGE and further transferred to the PVDF membrane. The membrane was incubated with appropriate antibodies, washed and incubated with HRP-labeled secondary antibodies, and then the blots were visualized using the ECL+plus Western Blotting Detection System (Amersham). The blots were quantitated by IMAGEQUANT, and the results were normalized by β-actin.

### Luciferase reporter assay

1.0×10^5^ cells were seeded in a 6-well plate with completed medium to grow until they reached 80% confluence. The related luciferase reporter plasmids (3μg) and 0.2μg pRL-CMV-Luc *Renilla* plasmid (from Promega) were transiently cotransfected, and in some experiments, the siRNA oligoneucleotides were cotransfected. After treatment, the cells were harvested and the luciferase activity assays were carried out using the Dual-Luciferase™ Assay System (Promega), and the transfection efficiencies were normalized using a cotransfected *Renilla* plasmid per manufacturers’ instructions.

### DNA methylation analysis

A real-time PCR based method for methylation specific PCR (MSP) analysis was used to evaluate DNA methylation on the human AHR promoter according to the previously described method [29]. The genomic DNA from treated cells was extracted and purified, and then treated by bisulfite modification using the EpiJET Bisulfite Conversion Kit (#K1461, Fisher). The modified DNA was then amplified using methylated and unmethylated primers for MSP with below details: Methylated primer Forward 5’- GGT TGG GGA GTT TCG TCG AC -3’, Reverse 5’- CCG CCT ACG AAA CTC GAA -3’; Unmethylated primer Forward 5’- GGT TGG GGA GTT TTG TTG AT -3’; Reverse 5’- CTT CCC ACC TAC AAA ACT CAA AC -3’. The final methylation readout was normalized by unmethylated input PCR, the PCR products were confirmed by electrophorese using 2% agarose gel, and the DNA bands were imaged.

### Chromatin immunoprecipitation (ChIP)

Cells were washed and crosslinked using 1% formaldehyde for 20 min and terminated by 0.1M glycine. Cell lysates were sonicated and centrifuged. 500μg of protein were pre-cleared by BSA/salmon sperm DNA with preimmune IgG and a slurry of protein A agarose beads. Immunoprecipitations were performed with the indicated antibodies, BSA/salmon sperm DNA and a 50% slurry of protein A agarose beads. Input and immunoprecipitates were washed and eluted, and then incubated with 0.2 mg/ml Proteinase K for 2h at 42°C, followed by 6h at 65°C to reverse the formaldehyde crosslinking. DNA fragments were recovered by phenol/chloroform extraction and ethanol precipitation. A 140-160bp fragment on either AHR or VEGF promoter was amplified by real-time PCR (qPCR) using the primers indicated in Table 1.

### Measurement of ROS generation

Treated cells were seeded in a 24-well plate and incubated with 10μM CM-H2DCFDA (Invitrogen) for 45 min at 37°C, and then the intracellular formation of reactive oxygen species (ROS) was measured at excitation/emission wavelengths of 485/530 nm using a FLx800 microplate fluorescence reader (Bio-Tek). The data was normalized as arbitrary units [46]. Levels of oxidative marker 3-nitrotyrosine (3-NT) were measured by western blots.

### Measurement of DNA breaks

8-OHdG formation was measured using an OxiSelect™ Oxidative DNA Damage ELISA Kit (Cat No. STA320, from Cell Biolabs Inc.) per manufacturers’ instructions. The formation of γH2AX was measured from nuclear extracts by western blotting using H2AX as the input control.

### Measurement of apoptosis

Apoptosis was evaluated by TUNEL assay using the *In Situ* Cell Death Detection Kit™ (Roche). Cells were fixed in 4% paraformaldehyde and labeled by TUNEL reagents. Stained cells were photographed by a fluorescence microscope and further quantified by FACS analysis. Caspase-3 activity was determined by the ApoAlert caspase assay kit (Clontech). Treated cells were harvested and 50 μg of proteins were incubated with the fluorogenic peptide substrate Ac-DEVD-7-amino-4-trifluoromethyl coumarin (AFC). The initial rate of free AFC release was measured using a FLx800 microplate reader (Bio-Tek) at excitation/emission wavelengths of 380/505 nm, and enzyme activity was calculated as pmol/min/mg [46].

### Measurement of mitochondrial function

The intracellular ATP level was determined by the luciferin/luciferase-induced bioluminescence system. An ATP standard curve was generated at concentrations of 10^-12^-10^-3^M. Intracellular ATP levels were calculated and expressed as nmol/mg protein. The mitochondrial membrane potential (Δψm) was measured by TMRE (from Molecular Probes T-669) staining. A 600μM T-669 stock solution was prepared using DMSO. Cells were grown on coverslips and immersed in 600 nM TMRE for 20 min at 37°C to load them with dye. The labeling medium was then aspirated and the cells were immersed in 150 nM TMRE to maintain the equilibrium distribution of the fluorophore. The coverslips were mounted with live cells onto confocal microscopes to image the cells using 548 nm excitation/573 nm emission filters. The intensity of TMRE fluorescence was measured using Image J software. Data from 10-20 cells were collected for each experimental condition and mean values of fluorescence intensity ± SEM were calculated [47].

### Cell viability and MTT assay

Cells were pooled in 12-well plates, following exposure to different treatments as indicated at 80% confluence. Cell viability was analyzed by the MTT (3-(4,5-dimethylthianol-2-yl)- 2,5 diphenyltetrazolium bromide) reduction assay [48]. Briefly, cells in each well were aspirated and washed with PBS, and then 0.2 ml of 0.3 mg/ml MTT solution were added at 25°C for 3 h. Thereafter, the precipitated blue formazan product was extracted by incubating samples with 0.1ml 10% SDS (dissolved by 0.01M HCl) overnight at 37°C. The optical density (OD) of formazan concentrations was determined at 560 nm and the background was subtracted at 670 nm, then normalized by cell numbers, and expressed as OD/10^6^ cells.

### DNA synthesis by [3H]-thymidine incorporation

Cell proliferation was evaluated as the rate of DNA synthesis by [3H]-methylthymidine incorporation [49]. Cells were pooled in 24-well plates until they reached 80% confluence, and then the indicated chemicals were added and incubated for 24 hours. At the end of the treatment, cells were incubated with serum-free media containing ^3^H-methylthymidine (0.5 μCi/well) for 2 hours, then washed twice with PBS. Cellular DNA was precipitated by 10% trichloroacetic acid and solubilized with 0.4 M NaOH (0.5 ml/well). Incorporation of ^3^H-methylthymidine into DNA was measured in a scintillation counter and was determined as counts per minute (CPM).

### Colony formation in soft agar

This assay is a method for evaluating the ability of individual cell lines to grow in an anchorage-independent manner. Cells were resuspended in DMEM containing 5% FBS with 0.3% agarose and layered on top of 0.5% agarose in DMEM on 60-mm plates. 1000 cells were seeded in 60mm soft agar dishes for 30 days, the dishes were examined twice per week, and colonies that grew beyond 50mm in diameter were scored as positive. Each experiment was done in quadruplicate.

### Generation of SOD2/HIF1C lentivirus expression THP1 cells

The human SOD2 expression lentivirus was generated as described previously in our lab [15]. In order to generate human constitutive HIF1α (HIF1C) lentivirus, the human wild type HIF1α cDNA was obtained from Open Biosystems, and the HIF1α double mutants at P402(A) and P564(A) named as constitutive HIF1α (HIF1C) [25] were generated using the Site-directed Mutagenesis Kit from Promega. It was then amplified by PCR and subcloned into the pLVX-Puro vector (from Clontech) using restriction sites of BamH I and Xba1 with the following primers: Forward: 5’- tcga-ggatcc – atg gag ggc gcc ggc ggc gcg -3’ (BamH I) and Reverse: 5’- gcgc-tctaga- tca gtt aac ttg atc caa agc -3’ (Xba I), and the HIF1C or empty (CTL) lentivirus was expressed through Lenti-X™ Lentiviral Expression Systems (from Clontech) per manufacturers’ instructions. The virus for HIF1C and SOD2 expression, or related empty (EMP) virus was used to infect THP1 cells, the positive clones were selected by 10μg/ml puromycin, the single colony was picked up, and the expression efficiency for both SOD2 and HIF1C was confirmed by real time PCR and western blotting. The stable SOD2/HIF1C expression THP1 cells were used for *in vivo* mice xenograft tumor study.

### *In vivo* xenograft tumor study

The Balb/c athymic nude male mice (6 weeks old) were obtained from the Disease Prevention Center of Guangdong Province. All procedures involving mice were conducted in accordance with NIH regulations concerning the use and care of experimental animals, and were approved by the Institutional Animal Care and Use Committee (from Peking University Shenzhen Hospital). The 2×10^6^ viable THP1 or THP1 with SOD2/HIF1C lentivirus expression cells were washed, harvested in PBS, and then injected into the lateral tail vein in a volume of 0.1ml. After 2 days of the implantation of the primary xenograft, the mice were treated by 25 mg/kg of body mass of either BA (corn oil as vehicle) or CDM (0.1% sodium carboxyl methylcellulose as vehicle), or a combination of BA/CDM via oral gavage 3 times a week. The mice with tail vein injection of lentivirus-infected THP1 cells were separated into 5 groups (n=9). Group 1 (CTL): THP1 cells (empty lentivirus) plus treatment of chemical vehicle (corn oil + 0.1% sodium carboxyl methylcellulose); Group 2 (BA): THP1 cells (empty lentivirus) plus treatment of BA; Group 3 (CDM): THP1 cells (empty lentivirus) plus treatment of CDM; Group 4 (BA/CDM): THP1 cells (empty lentivirus) plus treatment of BA and CDM; Group 5 (BA/CDM/SOD2/HIF1C): THP1 cells (SOD2/HIF1C lentivirus) plus treatment of BA and CDM. Mice were monitored for changes in body weight and killed when values fell below 20% of their initial weight. The lungs from sacrificed mice were isolated and fixed in 10% formalin. The number of surface metastases per lung was determined under a dissecting microscope. Formalin-fixed, paraffin-embedded tumor tissue from the lungs were sectioned to 4mm thickness, and the histopathological analyses were performed with H&E staining. Images were taken using a Carl Zeiss MIRAX MIDI slide scanner, and analyses were performed using a 3DHISTECH Pannoramic Viewer. The tumor tissues were isolated for *in vivo* monitoring of superoxide anion release, and the gene expression of SOD2, AHR and VEGF from tumor tissues were measured by real time PCR for mRNA and Western Blotting for protein levels.

### *In vivo* superoxide release

The superoxide anion (O_2_^.-^) release from the tumor tissue was determined by a luminol-EDTA-Fe enhanced chemiluminescence (CL) system supplemented with DMSO-TBAC (Dimethyl sulfoxide-tetrabutyl-ammonium chloride) solution for extraction of released O_2_^.-^ from tissues as described previously [46]. The superoxide levels were calculated from the standard curve generated by the xanthine/xanthine oxidase reaction.

### Statistical analysis

The data was given as mean ± SEM, and all of the experiments were performed at least in quadruplicate unless otherwise indicated. The one-way ANOVA followed by the Bonferroni post hoc test was used to determine statistical significance of different groups, and the mouse survival curve was determined by Kaplan-Meier survival analysis using SPSS 22 software, and a *P* value < 0.05 was considered significant.

## Abbreviations

AHR: aryl hydrocarbon receptor
AML: acute myeloid leukemia
ARNT: aryl hydrocarbon receptor nuclear translocator
AZA: 5-aza-2’-deoxycitidine
BA: betulinic acid
CDM: chidamide
ChIP: chromatin Immunoprecipitation
EBV: Epstein-Barr virus
ETC: electron transport chain
HDAC: Histone deacetylase
HDACi: histone deacetylase inhibitors
HIF1α: hypoxia-inducible factor-1α
HIF1C: constitutive HIF1α
O_2_.-: superoxide anions
ROS: reactive oxygen species
Sp1: specificity protein 1
SOD2: manganese superoxide dismutase
